# Informal caregivers of persons with dementia, their use of and needs for specific professional support: a survey of the National Dementia Programme

**DOI:** 10.1186/1472-6955-9-9

**Published:** 2010-06-07

**Authors:** José M Peeters, Adriana PA Van Beek, Julie HCM Meerveld, Peter MM Spreeuwenberg, Anneke L Francke

**Affiliations:** 1 The Netherlands Institute for Health Services Research (NIVEL), Otterstraat, Utrecht, The Netherlands; 2 Dutch Alzheimer's Association, Kosterijland, Bunnik, The Netherlands; 3 Department of Public and Occupational Health, EMGO Institute for Health and Care Research (EMGO+) of the VU University Medical Center, Van der Boechorststraat, Amsterdam, The Netherlands

## Abstract

**Background:**

This paper describes both the use of and needs for informal caregivers of people with dementia, based on a questionnaire survey organized within the National Dementia Programme in the Netherlands. The National Dementia Programme is a quality collaborative of the Dutch Alzheimer's Association, the Institute of Quality of Healthcare (CBO) and the Knowledge Centre on Ageing (Vilans), instigated by the Ministry of Health, Welfare and Sport, to improve integrated care for people with dementia and their informal caregivers. The support needs of informal caregivers are important to improve caregiver well-being and delaying institutionalization of the person with dementia.

**Methods:**

In the period April 2006 - January 2007, the National Dementia Programme questionnaire was completed by 984 informal caregivers. Descriptive statistics were used to analyze the use of and needs for additional professional support by informal caregivers. Chi-square tests were used to assess the relationships between characteristics of the caregivers (spouses, sons/daughters, sons/daughters in-law) and support needs on one hand and to assess the relationship between the living situation of the person with dementia (living at home or living in a nursing home or home for the elderly) and support needs on the other hand.

**Results:**

Almost all informal caregivers (92.6%) received some professional support. However, two thirds (67.4%) indicated they had one or more needs for additional professional support. Informal caregivers often need additional professional advice about what to do when their relative is frightened, angry of confused. Spouses reported different needs than sons or daughters (in-law): spouses relatively often need emotional support and sons or daughters (in-law) more often need information and coordination of dementia care.

**Conclusions:**

Most of the informal caregivers report that they need additional information and advice, e.g. about how to cope with behavioral problems of their relative, about the progression of the illness trajectory, emotional support and coordination of dementia care. Future support programmes, e.g. in the field of case management, should address the specific needs of informal caregivers.

## Background

The increasing prevalence of dementia and its impact on those who care for persons with dementia are growing problems worldwide [1-5]. Relatives are often closely involved in the care for people with dementia when the patient is living at home [6], but also when they are admitted to a long-term care facility [7,8]. The progressive and often unpredictable nature of dementia poses enormous challenges to informal caregivers. Spouses and other close relatives often participate in a number of activities in the various stages of dementia, such as obtaining a correct diagnosis, finding out which treatments are possible (medication and psychosocial management) and managing behavioral problems in the person with dementia [9,10]. Informal caregivers of persons with dementia may have substantial needs for different kinds of professional support i.e. for a variety of services, depending on the problems they experience. Professional care services may assist informal caregivers in supporting the person with dementia at home or may give informal caregivers a break [11].

Research of Gaugler, Kane, Kane and Newcomer [12,13] indicated that meeting the support needs of informal caregivers are important to improve caregiver well-being and delaying institutionalization of the person with dementia. A recent study, however, showed a rather large number of unmet care needs in both persons with dementia and in their informal caregivers [14]. Most unmet needs were related to a lack of knowledge about the existing service offer, a threshold to using services and insufficient service offer.

Although in the Netherlands as well as in other Western European countries, services like professional home care, respite care, daycare, and meeting centres for persons with dementia and their informal caregivers exist [15], they are not always broadly used by the target group [16]. One reason may be that the care supply may not fit with the needs and problems of informal caregivers.

So, insight in informal caregivers' support needs is important and relevant for further improving dementia care in the future. The purpose of this paper is to describe the use of and needs for specific professional support, based on a survey in a large population of informal caregivers of persons with dementia. The survey was organized within the framework of the so called National Dementia Programme from 2005 till 2008. The Dutch Ministry of Health, Welfare and Sport ordered the Dutch Alzheimer's Association, the Institute of Quality of Healthcare (CBO) and the Knowledge Centre on Ageing (Vilans) to develop a nation-wide quality collaborative programme by generating needs-driven care within regional networks for comprehensive dementia care. The programme is now implemented almost nationwide (95%).

One of the first initiatives within the National Dementia Programme was to explore main problems which informal caregivers of people with dementia have to face. Based on literature study, interviews with professional caregivers and panel discussions with informal caregivers and people with dementia a list of 14 so called problem areas was described (see Additional file 1) [17].

The 14 problem areas cover all the different stages of dementia: from the early onset of the disease to admission to a care facility. It is important to mention that these areas do not occur consecutively from 1 up to and including 14, and that problems may occur simultaneously, in combinations that vary per person with dementia.

In this paper, we address the needs of informal caregivers for additional professional support, while also discussing the professional support they already receive. In addition, we describe the relationship between the needs for professional support and the specific background characteristics of the informal caregivers and of the persons with dementia.

The specific research questions addressed are:

1. To what extent do informal caregivers receive professional support in the care for their relative, and what types of professional support do they receive?

2. Do informal caregivers have a need for additional professional support? If so, for what types of professional support and to what extent?

3. Do specific types of informal caregivers need specific types of additional professional support?

In this paper the 'informal caregiver' is mainly the central caregiver: e.g. spouses, daughters, daughters in-law, sons and sons-in-law. Further, we define additional professional support as 'professional care or support, in addition to the professional support informal caregivers already receive, for example care by district nurses, general practitioners or social workers'.

## Methods

### Questionnaire

The Dutch Alzheimer's Association ordered the Netherlands Institute for Health Care Services Research (NIVEL) to develop a questionnaire focusing on informal caregivers' problems and support needs, related to the 14 problem areas formulated in the National Dementia Programme. The development trajectory of this questionnaire consisted of the following steps: (1) a literature search focusing on existent instruments regarding dementia care, (2) consultations of several experts in the field of dementia care and (3) focus group discussions with informal caregivers. A draft questionnaire was tested on comprehensibility and applicability among 15 informal caregivers in different regions of the Netherlands.

The core parts of the questionnaire measures from the perspective of the informal caregiver (see Additional file 2):

(1) 14 problem areas, subdivided in 30 questionnaire items on problems that are faced by persons with dementia and problems experienced by informal caregivers;

(2) the importance of these problem areas, according to the informal caregiver;

(3) the informal caregiver's needs for additional professional support (56 items) [18]. In addition, the questionnaire asked about background characteristics of the informal caregivers themselves (gender, age, living arrangements, relationship with the person with dementia, type, frequency and duration of the informal care) and about characteristics of the person they cared for (gender, age, living arrangements, since when has the person shown signs of dementia and the use of professional support). The items about perceived actual problems (see (1) above) and the importance of these problems (2), were formulated as a statement after which informal caregivers had to indicate whether they experienced these problems in the last two months ('yes', 'actually yes', 'actually no', 'no' or 'not applicable') and how important the problems were at the moment ('of paramount importance', 'very important', 'important' and 'not so important').

As said, the questionnaire also asks whether caregivers have needs for specific types of professional support. These needs (3) are expressed in a list of suggestions for support, each directly linked to a particular problem area.

The reliability of the questionnaire in terms of internal consistency (Cronbach's alpha) was high: a = 0.81, based on the questions on the informal caregivers' perceived importance of the 14 problem areas at this moment.

We also evaluated the questionnaire on the basis of a few additional questions for informal caregivers about the clarity of the questionnaire and the applicability of the questions on the informal caregivers' situation. This evaluation demonstrated that the questionnaire was comprehensible and applicable to informal caregiver of persons with dementia in various situations (living at home, in a home for the elderly or nursing home). Besides, the questionnaire appeared to be suitable to informal caregivers who take care of persons with dementia in different phases of the illness.

### Recruitment Method

The questionnaire was distributed amongst informal caregivers by volunteers of the Dutch Alzheimer's Association (executive partner in the National Dementia Programme). One of the strategies of the Dutch Alzheimer's Association is training volunteers in supporting persons with dementia and their informal caregivers, e.g. by providing information, advice, counseling and advocacy.

Informal caregivers were recruited by 120 volunteers trained within the National Dementia Programme, during several activities organized by the Dutch Alzheimer's Association in the period April 2006 - January 2007: "Alzheimer Cafés", information meetings, focus groups and support groups for informal care givers (of about 10-15 participants in each group). The volunteers based their recruitment and information strategies on the procedures described in the questionnaire manual [18]. Informal caregivers were asked to complete the questionnaire immediately or at home, after volunteers had provided structured verbal and written information about the aim and background of the study.

### Subjects

A total of 984 informal caregivers of persons with dementia completed the questionnaire. The volunteers of the Dutch Alzheimer's Association did not systematically register how many informal caregivers they approached for completing the questionnaire (estimations are about 5000 - 6000 informal caregivers approached), which hampers the calculation of a reliable response rate.

All parts of the Netherlands (south, north, middle and east) are represented in the research group. Background characteristics of the 984 informal caregivers and of the persons with dementia they care for are presented in table 1.

**Table 1:** Characteristics of informal caregivers and of people with dementia (Number (%)).

Characteristics of informal caregivers	**N = 984 (%)**^**a**^
*Relationship to the person with dementia*	
Spouse	490 (50.8)
Son/daughter (in-law)	372 (38.2)
Other relative	84 (8.7)
Friend, neighbor, acquaintance	23 (2.3)
*Gender*	
Male	277 (28.4)
Female	697 (71.6)
*Age categories (years)*	
Below 45	81 (8.2)
45 - 55	216 (21.9)
55 - 65	218 (22.2)
65 - 75	209 (21.2)
75 - 85	207 (21.1)
85 and above	53 (5.4)
*Frequency of the care*	
Daily	511 (54.8)
3 - 6 times a week	170 (18.3)
1 - 2 times a week	203 (21.9)
Less than once a week	36 (3.9)
Less than once a month	11 (1.1)
*Duration of caregiving*	
Less than 6 months	26 (2.7)
6 months to 1 year	100 (10.5)
1 - 2 years	177 (18.4)
2 - 3 years	186 (19.3)
3 - 4 years	140 (14.5)
4 - 5 years	113 (11.7)
More than 5 years	221 (22.9)
*Assistance*	
Informal caregiver receives no or little assistance from other relatives	486 (51.9)
Other informal caregivers essentially contribute to the care for the person with dementia	448 (48.1)

**Characteristics of persons with dementia**	**N = 984 (%)**

*Living arrangements*	
Living at home	631 (69.0)
Living in a nursing home or in a home for the elderly	299 (31.0)
*Gender*	
Male	415 (42.3)
Female	564 (57.7)
*Age categories (years)*	
Below 45	2 (0.3)
45 - 55	11 (1.0)
55 - 65	79 (8.0)
65 - 75	231 (23.5)
75 - 85	462 (47.0)
85 and above	199 (20.2)
*Duration of dementia symptoms*	
Less than 6 months	17 (1.7)
6 months - 1 year	61 (6.3)
1 - 2 years	156 (15.9)
2 - 3 years	197 (20.2)
3 - 4 years	132 (13.5)
4 - 5 years	148 (15.2)
More than 5 years	265 (27.2)

*Note: *N = 984. Informal caregivers mean age (in years) was 62.8; SD was 13.3 and the range was 16-101.

*Note: *N = 984. Persons with dementia mean age (in years) was 78.2; SD was 8.79 and the range was 42-101.

a. Do not all add up to 984, due to 'missings'.

### Statistical Analysis

For answering research questions 1 and 2 (about the actual professional support received and about the caregivers' needs for additional support) descriptive analyses were performed. To answer research question 3, we used chi-square tests to test the relationship between the specific types of informal caregivers and their needs for additional professional support and to test the relationship between the living situation of the person with dementia and the informal caregivers' needs. The criterion for statistical significance was p < 0.05.

SPSS version 14.0 for Windows was used for the descriptive statistics and the chi-square tests.

## Results

### Description of the study population

Half of the informal caregivers in our study were spouses of the person with dementia (50.8%); in about one third the son or daughter (in-law) is the caregiver (38.2%). About three quarters of the informal caregivers are female (71.6%). The mean age of the informal caregivers is 62.8 years: the youngest informal caregiver is 16 years old and the oldest is 101 years. Almost all the informal caregivers look after their spouse or parent at least once a week (95.0%). More than half of the informal caregivers look after the person with dementia every day (54.8%). In addition, half of the informal caregivers provided care to the person with dementia on their own, with no or only little assistance from other relatives or acquaintances (51.9%).

Most of the persons with dementia are living at home with their spouse or in the household with their son or daughter (69.0%; see Table 1), and one third of the persons with dementia live in a nursing home or home for the elderly (31.0%). Almost six out of ten persons with dementia were female (57.7%; see Table 1). The mean age is 78.2 years: the youngest person with dementia is 42 and the oldest is 101 years old.

### Use of professional care

We asked the informal caregivers to what extent they receive professional support in the care for their relative: 92.6% did receive some kind of support (see Table 2). Almost four out of ten (37.1%; not in table) informal caregivers of people with dementia who are living at home receive support with the physical care for their relative, got support with the house keeping (45.0%) or their relative received medical dementia care (29.5%). Almost two third of the people with dementia (63.6%) visit a day-care centre or a meeting centre for persons with dementia for social and emotional support.

**Table 2:** Use of professional support related to informal caregivers' needs for additional professional support (Number (%)).

Use of professional support and extent of informal caregivers' needs	Living at home n = 631	Living in a nursing home or home for the elderly n = 299	**Total **^**b**^ N = 984
*Use of professional support*			
No use of professional support	63 (10.0)	0 (0.0)	73 (7.4) ^b^
Use of professional support	568 (90.0)	299 (100.0)	911 (92.6)
Total	631 (100.0)	299 (100.0)	984 (100.0)
*Needs for additional professional support*			
No needs for additional professional support	169 (26.8)	132 (44.1)	321 (32.6)
One or more needs for additional professional support	462 (73.2)	167 (55.9)	663 (67.4)
Total	631 (100.0)	299 (100.0)	984 (100.0)

b. The total number of each subgroup does not add up to the total: under "total" we added the answers of caregivers who completed the questionnaire, but could not be classified because of the missing of information about the caregiving situation.

*Note: *N = 984 Informal caregiver mean number of additional professional support needs 4.9, SD was 6.6. and the range was 0-55.

### Needs for additional professional support

Although almost in all cases already professional support was being received (see Table 2), two thirds (67.4%) of the informal caregivers indicated that they had one of more needs for additional professional support. Informal caregivers relatively often report they need advice about what to do when their relative with dementia is frightened, angry or confused (28.8%; see Table 3). Almost one out of five informal caregivers report they need to learn how to cope with changes in the behavior of the person with dementia (18.6%) and that they need more information about the professional help and support available in the region (18.6%). However, the specific types of additional professional support needed vary for the total research group. Besides, there are significant differences between informal caregivers of persons with dementia living at home and informal caregivers of people with dementia who are living in a nursing home or home for the elderly. Informal caregivers of persons with dementia who are living at home more often need someone to look after their relative with dementia, how to cope with behavior problems of the person with dementia; they also need more information about the legal amends in case of admission of the persons with dementia and more information about professional help and support options in their region (see Table 3).

**Table 3:** Differences between types of informal caregivers' needs for additional professional support (Number (%)).

Needs for additional professional help	Spouses N = 490	Sons/Daughters (in- law) n = 372	p value	Living at home n = 631	Living in a nursing home or home for the elderly n = 299	Total N = 984	p value
*Advice*							
I need to learn what to do when the person I am caring for is frightened, angry or confused	**115** **(23.3)**	**133** **(35.8)**	0.001*	**182** **(28.8)**	**86** **(28.8)**	**282** **(28.8)**	**0.997**
I need to know how to deal with the apathy of the person I am caring for	**98** **(19.8)**	**69** **(18.6)**	**0.287**	**133** **(21.0)**	**38** **(12.7)**	**182** **(18.4)**	0.002*
I need to learn how to cope with changes in the behavior of the person I am caring for	**83** **(16.8)**	**83** **(22.3)**	**0.186**	**130** **(20.5)**	**43** **(14.4)**	**186** **(18.6)**	0.024*
I need to learn how to deal with the aggression of the person I am caring for	**66** **(13.4)**	**56** **(15.1)**	**0.755**	**83** **(13.1)**	**43** **(14.4)**	**138** **(13.5)**	**0.597**
I need to know which activities I can undertake with the person I am caring for	**66** **(13.4)**	**46** **(12.4)**	**0.894**	**88** **(13.9)**	**26** **(8.7)**	**124** **(12.2)**	0.024*

*Information*							
I need more information about the professional help and support available in the region	**90** **(18.2)**	**81** **(21.8)**	**0.093**	**150** **(23.7)**	**23** **(7.7)**	**183** **(18.6)**	0.000*
I need information about the progression of dementia	**68** **(13.8)**	**79** **(21.2)**	0.014*	**117** **(18.5)**	**44** **(14.7)**	**167** **(17.3)**	**0.156**
I need information about the legal amends in case of admission of the person with dementia to a nursing home or home for the elderly	**100** **(20.2)**	**41** **(11.0)**	0.002*	**131** **(20.7)**	**16** **(5.4)**	**155** ** (15.8)**	0.000*

*Coordination of dementia care*							
I want professional caregivers to work together and to tailor the care	**62** **(12.6)**	**75** **(20.2)**	0.005*	**104** **(16.4)**	**43** **(14.4)**	**156** **(15.8)**	**0.432**
I want to reflect the professional care from time to time with professional caregivers	**56** **(11.3)**	**67** **(18.0)**	0.050*	**89** **(14.1)**	**44** **(14.7)**	**139** **(14.3)**	**0.789**

*Emotional support*							
I need someone to take away the feeling that I am the only one to look after the person with dementia	**85** **(17.2)**	**38** **(10.2)**	0.024*	**95** **(15.0)**	**32** **(10.7)**	**135** **(13.6)**	**0.074**
I need someone to look after the person with dementia now and then	**74** **(15.0)**	**44** **(11.8)**	**0.399**	**109** **(17.2)**	**10** **(3.3)**	**129** **(12.8)**	0.000*
I need emotional support	**81** **(16.4)**	**38** **(10.2)**	0.016*	**87** **(13.8)**	**34** **(11.4)**	**129 (13.0)**	**0.314**

*Differences between the scores of 'spouses' and 'sons/daughters (in-law)'versus differences between 'living at home' and 'living in a nursing home or home for the elderly' are statistically significant (Chi-square test, p < 0.05).

The amount of additional professional support needed is also related to whether the person with dementia lives at home, in a nursing home, or home for the elderly. Almost three quarters (73.2%; see Table 2) of the informal caregivers of persons with dementia who are living at home have one or more kinds of need for additional professional support. This percentage is significantly lower for informal caregivers of dementia persons who are living in a nursing home or home for the elderly: 55.9% of these informal caregivers have one or more kinds of need for additional professional support (chi-square; p = 0.000).

### Specific needs for additional professional support: differences between spouses and sons or daughters (in-law)

We already noticed that informal caregivers relatively often need additional professional advice about what to do when their relative with dementia is frightened, angry or confused. In this regard there is a significant difference between spouses and sons or daughters in law: 23.3% of the spouses and 35.8% of the sons or daughters (in-law) in our study reported that they need more advice on how to handle such behavior problems (chi-square; p < 0.05; see Table 3). However, with regard to certain other support needs, there are more significant differences between spouses and sons or daughters (in-law). Spouses' needs often concern emotional support (16.4% of the spouses versus 10.2% of the sons or daughters (in-law)) and they report to need someone to take away the feeling that they are the only person to look after their relative (17.2% of the spouses versus 10.2% of the sons or daughters (in-law)). Sons or daughters (in-law) relatively often report that they would like more information about the progression of the illness dementia (21.2% of the sons or daughters (in-law) versus 13.8% of the spouses). They also have a relatively greater need for better coordination between professional support and informal care (20.2% of the sons or daughters (in-law) versus 12.6% of the spouses) and need to have more consultation with the professional caregivers (18.0% of the sons or daughters (in-law) versus 11.3% of the spouses). However, spouses (20.2%) more often than sons or daughters (in-law); 11.0%)) have a need for information about the legal amends when their relative is admitted in a nursing home or in a home for the elderly.

## Discussion

In our study 92.6% of the informal caregivers and their relatives with dementia received professional support. However, a large group remains in need of additional professional support: two thirds of the caregivers of persons with dementia (67.4%) need more professional support than they are currently receiving. The need for additional professional support most often concerns advice about how to deal with behavior problems in the person with dementia (28.8%). Spouses and sons or daughters (in-law) have specific needs for additional professional support. Spouses often require emotional support and respite care, while sons or daughters (in-law) mainly need more information about the progression of dementia. Sons or daughters (in-law) of persons with dementia staying in a nursing home or in a home for the elderly relatively often need additional consultations with professional caregivers and coordination of dementia care.

It is important to add that the informal caregivers' needs for additional support vary and that most needs are mentioned by less than 20% of the informal caregivers. But, when we take into account that the vast majority of the informal caregivers in our study already use professional care, these percentages of needs can be considered as rather high. This implicates that informal caregivers have specific needs which are not met at the moment by their professional caregivers.

Our research findings are in line with previous research showing that caregivers of people with dementia have substantial needs for professional support [14,19,20]. But, the results of our large-scale study also provide insight into differences in needs between various types of informal caregivers. This study indicates that in order to achieve customized support for informal caregivers, the difference in needs of various types of central informal caregivers (e.g. spouses versus sons/daughters (in-law)) and the living situation of the people with dementia (living at home versus residential elderly care) must be taken into account.

### Strengths and limitations

The sample of our study is large and represents informal caregivers of persons with dementia in various settings (at home, in nursing homes or in homes for the elderly). Main characteristics of the persons with dementia who were cared for by the informal caregivers in our sample appear to be in line with characteristics mentioned in a prominent report of the Health Council of the Netherlands [21]. This report indicates that about 65% of the persons with dementia lived at home, while the other 35% lived in a nursing home or in a home for the elderly (in our study 69.0% versus 31.0% respectively). According to the Health Council, the mean age of persons with dementia is about 79 year (in our study 78.2 years).

A limitation of our study is, however, that we do not know how representative the investigated informal caregivers themselves were. The fact that the informal caregivers who completed the questionnaire were recruited at information meetings, focus groups or support groups may have caused selection bias. The way of recruitment implies that all informal caregivers participating in our study are already acquainted with a certain level of support. Findings may therefore not be generalized in all regards to informal caregivers who do not yet take part in activities such as information meetings, focus groups or support groups.

On the other hand, the sample of our study is large (N = 984) and represents informal caregivers of persons with dementia in different settings (at home, in nursing homes or home for the elderly) and also involves different types of informal caregivers e.g. spouses, daughters or sons (in-law) and other relatives. Therefore the results may be relevant for informal caregivers in different situations.

## Conclusions

Most of the informal caregivers in our study report that they need additional information and advice, e.g. about how to cope with behavioral problems of their relative with dementia and about the development of the illness trajectory. This finding stimulated the Dutch Alzheimer's Association and other national partners involved in the National Dementia Programme, to develop a plan for the structural national implementation of case management in dementia. The expectation is that support by a case manager (often a nurse specialized in dementia care) would particularly meet the informal caregivers' needs for professional advice and information. In addition, it is expected that case management would satisfy the need for better coordination between professional support and informal care. However, until now there has been little evidence of the long term effects of case management, for example in reducing or preventing caregivers' problems [22-24]. So, future research is needed in this regard.

## Competing interests

The Dutch Alzheimer's Association supported the study financially.

## Authors' contributions

JP conducted the conception and design of the study, data analysis and interpretation on the data, drafting and preparation of the manuscript. AB conducted the conception and design of the study, the interpretation of the data and consultation. JM did the acquisition of subjects and commentated on the findings of the study. PS gave methodological advice and performs the multi level analyses. AF contributed to the conception and design of the study and the manuscript, supervision of analysis of the data and drafting the manuscript. AB and AF reviewed the paper. All authors read and approved the final manuscript.

## Pre-publication history

The pre-publication history for this paper can be accessed here:

http://www.biomedcentral.com/1472-6955/9/9/prepub

## Supplementary Material

Additional file 1 **The 14 problem areas of the National Dementia Programme**. Description of the 14 so-called problem areas of the National Dementia Programme. Click here for file

Additional file 2 **Questionnaire 'National Dementia Programme Survey'**. Click here for file
