# Supplementary material for: Informal caregivers of persons with dementia, their use of and needs for specific professional support: a survey of the National Dementia Programme
Source: BMC Nurs. 2010 Jun 7;9:9. doi: 10.1186/1472-6955-9-9 (PMC2901350; doi:10.1186/1472-6955-9-9)
Supplement: Additional file 1 — The 14 problem areas of the National Dementia Programme. Description of the 14 so-called problem areas of the National Dementia Programme. [file 1472-6955-9-9-S1.PDF]

The 14 problem areas of the National Dementia Programme

National Dementia Programme – Problem areas

1. **Feeling that 'something is wrong'**
   There is a feeling of discomfort in the first phase of dementia about what is going on. People suspect dementia, but the feeling is vague.
2. **What is the matter and what might help?**
   After the diagnose dementia, the person with dementia and their caregivers do not know how to deal with the daily problems.
3. **Frightened, angry and confused**
   The person with dementia could have all kinds of behavior problems, such as angriness, suspicion or apathy.
   This can cause problems for the person with dementia and also for the informal caregiver.
4. **Having to cope all alone**
   The person with dementia is not capable of acting or does not remember were to find his/her belongings.
   The informal caregiver is not used to take care, and does not know where to find help.
5. **Avoiding contacts**
   The entrance with others is getting worse because of problem behavior of the person with dementia.
   The social network is getting smaller. The informal caregiver feels forced to stay at home.
6. **Physical care**
   The person with dementia needs help with dressing, showering, eating and dealing with incontinence.
7. **Dangers**
   This problem area concerns dangers for accidents in or around the house. The person with dementia is forgetful, defenseless, restless and wanders. The informal caregiver is very worried.
8. **Health problems too**
   The person with dementia forgets to take his/her pills and does not comply with medical treatment.
   He/she is becoming confused because of the medical problems.
9. **Loss**
   The person with dementia is getting worse, physical as well as mentally and is losing the grip on his/her life. This process goes together with denial, sadness and depression. The informal caregiver is mourning and feels lonely.
10. **Feeling overwhelmed**
    The informal caregiver is developing emotional problems and physical burden.
11. **Reduced say in matters, no say at all**
    To lose control is mainly a problem of the informal caregiver. He/she feels patronized by the formal caregiver.
12. **In good times and bad**
    Informal caregivers feel obliged to take care of the person with dementia. He/she feels guilty and is ashamed of the dedication of formal caregivers.
13. **Miscommunication with care workers**
    This problem area concerns the miscommunication and lack of connection of the person with dementia and their informal caregivers on one hand, and the formal caregivers on the other hand.
14. **Resistance to admission**
    The person with dementia and their informal caregiver may shrink for admission in a nursing home of home for the elderly. The admission is getting delayed or the informal caregiver is looking for alternatives.
