# Supplementary material for: Informal caregivers of persons with dementia, their use of and needs for specific professional support: a survey of the National Dementia Programme
Source: BMC Nurs. 2010 Jun 7;9:9. doi: 10.1186/1472-6955-9-9 (PMC2901350; doi:10.1186/1472-6955-9-9)
Supplement: Additional file 2 — Questionnaire 'National Dementia Programme Survey'. [file 1472-6955-9-9-S2.PDF]

NIVEL
The Netherlands Institute
for Health Services Research

Otterstraat 118-124
P.O.Box 1568
3500 BN Utrecht
The Netherlands
Telefoon +31 302 729 700

Questionnaire

National Dementia Programme Survey
Needs and problems of informal caregivers of persons with dementia

---

This questionnaire concerns the problems that you, as a caregiver of a person with dementia, may experience and the support that you would like to receive for these problems. The questionnaire was developed by NIVEL (the Netherlands Institute for Health Services Research) on behalf of the Dutch Alzheimer's Association.

The aim of this questionnaire is to gain insight into both the problems experienced by caregivers in caring for a person with dementia, and the support that they as caregivers would like to receive. The results of the questionnaire will be used to tailor better to the needs of caregivers.

This questionnaire deals with a range of problems. You may possibly recognise all the problems and needs. It may also be possible that you have not (yet) encountered certain problems. All the questions ask for **your own opinion**. This means you can never give a 'wrong' answer: it's your opinion that matters. **In this questionnaire, the relative with dementia that you are caring for is known as 'the person you are caring for'.**

The questionnaire addresses the period of the past two months. It starts with a number of questions about yourself and the person you are caring for. These details help us to create a picture of the problems experienced by informal caregivers. You are then presented with a number of problems and are asked to rate the importance of these issues for you currently. In the last part of the questionnaire you can indicate the areas in which you would like to receive more professional support.

All your data will be treated confidentially and anonymously. The results of this questionnaire will never be traceable to the individuals who have provided the answers. It takes about **30 minutes** to complete the questionnaire. If you have any questions, you can always address these to the volunteer of the Dutch Alzheimer's Association who has handed you the questionnaire.

NIVEL, April 2006

Date:
Municipality:
Region:
(to be filled in by a volunteer of the Dutch Alzheimer's Association)

A. General questions about yourself

Below are some questions about you and the person you are caring for. For each question you should mark one box, unless indicated otherwise.

1. Are you male or female?
   - ❑ Male
   - ❑ Female

2. What is your age? . . . Years

3. What is your civil status?
   - ❑ Unmarried
   - ❑ Married
   - ❑ Widowed
   - ❑ Divorced/separated

4. What is the highest education level you have completed?
   - ❑ No schooling
   - ❑ Primary school
   - ❑ Lower secondary school or similar
   - ❑ Higher secondary school
   - ❑ Middle vocational education
   - ❑ Third level vocational education
   - ❑ University education
   - ❑ Not known

5. What is your living situation?
   - ❑ Living alone
   - ❑ Living with a partner
   - ❑ Living with a partner and children

6. What is your residential situation?
   - ❑ Living at home
   - ❑ Living in a son's or daughter's home
   - ❑ Living in a care institution
   - ❑ Other, namely: ....

     ....

7. What is your relationship to the person with dementia?
   - ❑ Partner
   - ❑ Son/daughter (in-law)
   - ❑ Relative
   - ❑ Neighbor
   - ❑ Other, namely: ....

     ....

8. For how long have you been looking after the person since the first signs of dementia appeared (approximately)?
   - ❑ Less than half a year
   - ❑ Half a year to 1 year
   - ❑ 1 to 2 years
   - ❑ 2 to 3 years
   - ❑ 3 to 4 years
   - ❑ 4 to 5 years
   - ❑ More than 5 years

9. How many times per week do you provide care for the person with dementia?
   - ❑ Daily
   - ❑ 3 to 6 times per week
   - ❑ 1 to 2 times per week
   - ❑ Less than once per week
   - ❑ Less than once per month

10. Are there other relatives/friends who have a significant share in the care?
    - ❑ No, I am the main caregiver
    - ❑ Yes, one other person
    - ❑ Yes, two others
    - ❑ Yes, three or more others

11. What activities do you help with?
    (more than one answer possible)
    - ❑ Washing and/or dressing/undressing
    - ❑ Showering
    - ❑ Taking medication
    - ❑ Eating meals
    - ❑ Walking, standing
    - ❑ Household tasks
    - ❑ Cooking, food preparation
    - ❑ Transport, driving
    - ❑ Shopping
    - ❑ Administration
    - ❑ External activities (such as in a club or association)
    - ❑ Odd jobs (including cleaning, gardening, repairs, maintenance)
    - ❑ Coping with fears, anger or confusion
    - ❑ Other, namely: ....

      ....

General questions about the person you are caring for

1. Is the person with dementia male or female?
   - ❑ Male
   - ❑ Female

2. What is his/her age? . . . Years

3. What is his/her civil status?
   - ❑ Unmarried
   - ❑ Married
   - ❑ Widowed
   - ❑ Divorced/separated

4. What is his/her residential situation?
   - ❑ Living at home
   - ❑ Living in a son's or daughter's home
   - ❑ Living in a care institution
   - ❑ Other, namely: ....

     ....

5. What is the highest education level that he/she has completed?
   - ❑ No schooling
   - ❑ Primary school
   - ❑ Lower secondary school or similar
   - ❑ Higher secondary school
   - ❑ Middle vocational education
   - ❑ Third level vocational education
   - ❑ University education
   - ❑ Not known

6. Since when has the person shown signs of dementia (approximately)?
   - ❑ Less than half a year
   - ❑ Half to 1 year
   - ❑ 1 to 2 years
   - ❑ 2 to 3 years
   - ❑ 3 to 4 years
   - ❑ 4 to 5 years
   - ❑ More than 5 years

7. What professional help is currently being received by the person you are caring for?
   - ❑ No professional help
   - ❑ Physical care/nursing
   - ❑ Support with household tasks
   - ❑ Talks with a counsellor or advisor for the elderly or persons with dementia etc.
   - ❑ Day care treatment/Day care centre/ Social encounter centre
   - ❑ Medical treatment
   - ❑ Other, namely: ....

     ....

B. Problems that you may experience

Below are some questions about the problems that you may experience. Please tick the boxes that are relevant to **YOU**.

**Feeling that 'something is wrong' & What is the matter and what might help?**	**yes**	**actually yes**	**actually no**	**no**	**n.a.**
1. Did your GP take your first suspicions of dementia seriously?	❑	❑	❑	❑	❑
2. Was the explanation of dementia and its symptoms sufficient for you?	❑	❑	❑	❑	❑
3. Was the explanation of the progression of dementia sufficient for you?	❑	❑	❑	❑	❑

B1.How important are the above problems to you currently?
- ❑ Of paramount importance
- ❑ Very important
- ❑ Important
- ❑ Not so important

The following questions concern the past two months

**Frightened, angry and confused**	**yes**	**actually yes**	**actually no**	**no**	**n.a.**
4. Do changes in the person's behavior trouble you?	❑	❑	❑	❑	❑
5. Do you know how to handle the person with dementia when he/she is scared, angry or confused?	❑	❑	❑	❑	❑

B2.How important are the above problems to you currently?
- ❑ Of paramount importance
- ❑ Very important
- ❑ Important
- ❑ Not so important

Having to cope all alone	yes	actually yes	actuall y no	no	n.a.
6. Is there someone you can turn to with your questions or problems?	❑	❑	❑	❑	❑
7. Do you have the feeling that you are on your own?	❑	❑	❑	❑	❑
8. Can you offer sufficient help with daily tasks to the person you are caring for?	❑	❑	❑	❑	❑

B3. How important are the above problems to you currently?
- ❑ Of paramount importance
- ❑ Very important
- ❑ Important
- ❑ Not so important

Physical care	yes	actually yes	actuall y no	no	n.a.
9. Can you offer sufficient physical care to the person you are caring for?	❑	❑	❑	❑	❑
10. Does the person receive sufficient physical care from professional carers?	❑	❑	❑	❑	❑

B4. How important are the above problems to you currently?
- ❑ Of paramount importance
- ❑ Very important
- ❑ Important
- ❑ Not so important

Avoiding contacts	yes	actually yes	actually no	no	n.a.
11. Do you avoid contact with your family because of the dementia of the person you are caring for?	❑	❑	❑	❑	❑
12. Do you avoid contact with your friends because of the dementia of the person you are caring for?	❑	❑	❑	❑	❑

B5. How important are the above problems to you currently?
- ❑ Of paramount importance
- ❑ Very important
- ❑ Important
- ❑ Not so important

Dangers	yes	actually yes	actuall y no	no	n.a.
13. Are you worried that unsafe situations might occur in or around the house because of the person's behavior?	❑	❑	❑	❑	❑

B6. How important are the above problems to you currently?

- ❑ Of paramount importance
- ❑ Very important
- ❑ Important
- ❑ Not so important

Health problems too	yes	actually yes	actuall y no	no	n.a.
14. Does anyone keep an eye on the person's medicine intake, and check to what extent the person with dementia keeps to the recommended treatment?	❑	❑	❑	❑	❑
15. Do you think that health problems occur because the person with dementia has difficulty indicating physical ailments?	❑	❑	❑	❑	❑

B7. How important are the above problems to you currently?

- ❑ Of paramount importance
- ❑ Very important
- ❑ Important
- ❑ Not so important

Loss	yes	actually yes	actuall y no	no	n.a.
16. Can you cope with your grief about the deterioration in the person you are caring for?	❑	❑	❑	❑	❑
17. Do you feel lonely?	❑	❑	❑	❑	❑

B8. How important are the above problems to you currently?

- ❑ Of paramount importance
- ❑ Very important
- ❑ Important
- ❑ Not so important

**Feeling overwhelmed**	**yes**	**actually yes**	**actually no**	**no**	**n.a.**
18. Is the care for the person with dementia often too much of a burden for you physically?	❑	❑	❑	❑	❑
19. Is the care for the person with dementia often too much of a burden for you emotionally?	❑	❑	❑	❑	❑
20. Do you have less contact with relatives because of the person's illness?	❑	❑	❑	❑	❑
21. Do you have less contact with friends/acquaintances or less contacts in clubs because of the person's illness?	❑	❑	❑	❑	❑

B9. How important are the above problems to you currently?

- ❑ Of paramount importance
- ❑ Very important
- ❑ Important
- ❑ Not so important

**Reduced say in matters, no say at all**	**yes**	**actually yes**	**actually no**	**no**	**n.a.**
22. Do care workers involve you sufficiently in important decisions?	❑	❑	❑	❑	❑
23. Do you feel patronized by care workers?	❑	❑	❑	❑	❑
24. Do you find it difficult having to make decisions for the person you are caring for?	❑	❑	❑	❑	❑

B10. How important are the above problems to you currently?

- ❑ Of paramount importance
- ❑ Very important
- ❑ Important
- ❑ Not so important

In good times and bad	yes	actually yes	actually no	no	n.a
25. Do you feel guilty whenever you delegate care to care workers?	❑	❑	❑	❑	❑
26. Do you feel ashamed whenever you delegate care to care workers?	❑	❑	❑	❑	❑

B11.How important are the above problems to you currently?

- ❑ Of paramount importance
- ❑ Very important
- ❑ Important
- ❑ Not so important

Miscommunication with care workers	yes	actually yes	actually no	no	n.a.
27. Does the professional care or help you receive fit in with your wishes?	❑	❑	❑	❑	❑
28. Do you think that the care workers pay enough attention to you?	❑	❑	❑	❑	❑

B12.How important are the above problems to you currently?

- ❑ Of paramount importance
- ❑ Very important
- ❑ Important
- ❑ Not so important

Resistance to admission	yes	actually yes	actually no	no	n.a.
29. Do you shrink from having the person you are caring for admitted to a care institution?	❑	❑	❑	❑	❑
30. Do you have sufficient support in the case of a possible admission of the person you are caring for?	❑	❑	❑	❑	❑

B13.How important are the above problems to you currently?

- ❑ Of paramount importance
- ❑ Very important
- ❑ Important
- ❑ Not so important

C. Your needs for more support

Do you need **more** help or support than you are currently receiving? By this is meant the need for **professional help only.** This includes, for instance, district nursing, a day care centre, GP or medical specialist. More than one answer is possible. But if you don't want more help, you don't need to tick any boxes.

Feeling that 'something is wrong' & What is the matter and what might help
Tick only where you need more help
- ❑ I need support in setting up help
- ❑ I want the dementia to be firmly diagnosed
- ❑ I want information about the symptoms of dementia
- ❑ I want information about the course of dementia
- ❑ I want information about possible medical treatment of dementia
- ❑ I want to know what care/help I can get in my neighbourhood

Frightened, angry and confused
Tick only where you need more help
- ❑ I need help in learning how to cope with changes in the behavior of the person I am caring for
- ❑ I want to know how I can cope with apathy or lack of activity in the person I am caring for
- ❑ I want to know how I can handle aggression in the person I am caring for
- ❑ I want to know what I can do whenever the person is afraid, angry or confused

Having to cope all alone
Tick only where you need more help
- ❑ I want help with household chores
- ❑ I want help with preparing food and/or cooking for the person I am caring for
- ❑ I want help with transport for the person I am caring for, for example someone who can drive a car
- ❑ I want help with shopping for the person I am caring for
- ❑ I want help with the administration of the person I am caring for
- ❑ I want help with odd jobs (for example, maintenance, repairs, the garden)
- ❑ I need someone who can take away my feeling of being on my own in looking after the person I am caring for

Physical care
Tick only where you need more help
- ❑ I want help with washing and dressing/undressing the person I am caring for
- ❑ I want help with showering the person I am caring for
- ❑ I want help when the person I am caring for is eating
- ❑ I want help for the person I am caring for when he/she walks and stands up
- ❑ I want help to prevent the person I am caring for from falling
- ❑ I want help for incontinence in the person I am caring for

Avoiding contacts
Tick only where you need more help
- ❑ I want to know how I can undertake activities with the person I am caring for
- ❑ I want help not to feel ashamed about the behavior of the person I am caring for
- ❑ I want help in order to enable my family and friends to understand the behavior of the person I am caring for

Dangers

Tick only where you need more help

- ❑ I want help to prevent the person I am caring for from wandering off
- ❑ I want help to prevent unsafe situations
- ❑ I want help with making adaptations to the house
- ❑ I want help to prevent fires
- ❑ I want help in learning to cope with my anxiousness

Health problems too

Tick only where you need more help

- ❑ I want someone to keep an eye on the medication intake of the person I am caring for
- ❑ I want someone to check that the person I am caring for follows the recommended treatment
- ❑ I want help in identifying health problems in the person I am caring for

Loss

Tick only where you need more help

- ❑ I want help in coping with my grief about the dementia of the person I am caring for
- ❑ I want help in coping with my feelings of loneliness

Feeling overwhelmed

Tick only where you need more help

- ❑ I want help in sharing the care with family and friends
- ❑ I want someone to take over, once in a while, the support, help and care of the person I am caring for
- ❑ I want help in making a practical daily schedule
- ❑ I want emotional support

Reduced say in matters, no say at all

Tick only where you need more help

- ❑ I want the care providers to involve me in decisions
- ❑ I want help in making arrangements to become a mentor (mentor = taking decisions formally for the person cared for, about care, nursing, treatment and support)
- ❑ I want help with removing the driver's licence of the person I am caring for

In good times and bad

Tick only where you need more help

- ❑ I don't want to be patronized by care providers
- ❑ I want help in getting rid of my feelings of guilt about delegating the care to others (for example when the person I am caring for goes to a day care centre)
- ❑ I want help because the care is too much of a burden to me, I cannot cope any longer

Miscommunication with care workers

Tick only where you need more help

- ❑ I want to have discussion opportunities with professional care workers
- ❑ I want professional care workers to consider my wishes
- ❑ I want professional care workers to collaborate and to mutually ensure proper coordination of care
- ❑ I want help with the coordination of the care with other non-professional carers

Resistance to admission
Tick only where you need more help

- ❑ I want help in discussing admission to a care institution with the person I am caring for
- ❑ I want information about possible care institutions
- ❑ I want help to enable me to accept that admission of the person I am caring for to a care institution is necessary
- ❑ I want information about alternatives to admission to a care institution
- ❑ I want help, support and coaching instead of sending the person I am caring for to a care institution
- ❑ I want to know the legal regulations/financial compensation that apply to admission to a care institution

Other

- ❑ I want other help, i.e. (please fill in here what kind of professional help you would like to receive):

....

Space for remarks

....

....

....

....

....

....

....

Evaluation of the questionnaire

We would like to know if you think the questionnaire could be improved. The following questions seek your opinion on this. Please tick the boxes that apply to you.

1. Approximately how long did it take you to complete the questionnaire?
   - ❑ 0 to 15 minutes
   - ❑ 15 to 30 minutes
   - ❑ 30 to 40 minutes
   - ❑ More than 40 minutes

2. How did you find the length of the questionnaire?
   - ❑ Short
   - ❑ Just right
   - ❑ Too long

3. Did you find the questions clear?
   - ❑ Yes
   - ❑ No
   - ❑ Don't know

4. How do you rate the written explanations to the questions?
   - ❑ Good
   - ❑ Reasonable
   - ❑ Inadequate
   - ❑ Bad

5. Have these questions managed to produce a clear picture of your situation?
   - ❑ Yes
   - ❑ No
   - ❑ Don't know

6. The questionnaire concerned only the past two months. Does this period represent a good overview of your situation?
   - ❑ Yes
   - ❑ No
   - ❑ Don't know

MANY THANKS FOR COMPLETING THIS QUESTIONNAIRE
